# Editorial: Panoramic view of cognitive impairment: interdisciplinary cognitive research, volume II

**DOI:** 10.3389/fnins.2026.1988547

**Published:** 2026-09-25

**Authors:** Chong Tian, Fangyi Xu, Yang Xiao, Ran Wang

**Affiliations:** 1 School of Nursing, Tongji Medical College, Huazhong University of Science and Technology, Wuhan, China; 2 University of Louisville, Louisville, KY, United States; 3 Department of Central Laboratory, The Affiliated Huai'an No. 1 People's Hospital of Nanjing Medical University, Huai'an, China; 4 National Key Laboratory of Multispectral Information Intelligent Processing Technology, School of Artificial Intelligence and Automation, Huazhong University of Science and Technology, Wuhan, China; 5 Department of Communication, School of Journalism and Information Communication, Huazhong University of Science and Technology, Wuhan, China

**Keywords:** cognition, cognitive impairment, cognitive phenotype, interdisciplinary research, methodology

Cognitive impairment encompasses a broad spectrum, from subjective cognitive complaints and mild cognitive impairment to major neurocognitive disorders and dementia. As populations age, it has become a major public health concern, affecting not only cognitive performance but also functional independence, wellbeing, and quality of life. Despite advances in understanding biological and clinical underpinnings, challenges remain in risk identification, trajectory prediction, accessible assessment, and effective prevention and rehabilitation.

The multifactorial nature of cognitive impairment demands interdisciplinary perspectives. The first volume of this Research Topic covered risk factors, biomarkers, lifestyle influences, sensory-cognitive relationships, gait-based assessment, and therapies (Tian et al., 2024). Volume II extends this by linking cognitive impairment to broader clinical contexts, environmental and molecular mechanisms, functional autonomy, emerging assessment technologies, and multidimensional interventions.

Several contributions address populations with neurological or systemic vulnerability. Two systematic reviews examined post-stroke cognitive impairment from complementary angles. One meta-analysis estimated that about 46% of individuals experience impairment after a first stroke, with variation by sex, age, subtype, and vascular comorbidities (Zhang X. et al.). Another systematic review focused on cognitive frailty among older Chinese stroke patients, highlighting the co-occurrence of cognitive and physical vulnerability (Li et al.). Together, they underscore the need for cognitive assessment in comprehensive stroke care.

Volume II also extends across the life course beyond classical neurodegenerative disorders. An EEG study in children with attention deficit/hyperactivity disorder showed that electrophysiological measures can complement behavioral assessments to evaluate intervention effects (Khachidze et al.). In cervical dystonia, GDF15 levels were elevated but not consistently associated with global cognition, indicating the exploratory nature of this biomarker (Drużd et al.). Iatrogenic vulnerability is also addressed, a rat study demonstrated that the CMF chemotherapy regimen induced marked cognitive deficits across spatial, object recognition, and fear conditioning tasks, accompanied by reduced mitochondrial complex I activity, elevated reactive oxygen species, and increased inflammatory cytokines. Co-treatment with pioglitazone, a PPAR-γ agonist, failed to ameliorate these effects, highlighting the treatment-resistant nature of chemotherapy-related cognitive impairment and the need for further mechanistic research (Alhowail).

Environmental and molecular dimensions are further explored. A review of aryl hydrocarbon receptor (AhR) signaling describes this pathway as an interface between environmental chemical exposure and host-microbiome metabolism, contrasting neurotoxic effects of environmental polycyclic aromatic hydrocarbons with potentially neuroprotective signaling from microbiota-derived tryptophan metabolites (Tian et al.). Another study on survivors of toxic oil syndrome, four decades after exposure, used eye-tracking and neuropsychological tests to reveal antisaccadic abnormalities and selective deficits in executive function, attention, and processing speed, consistent with persistent frontal-subcortical dysfunction (Benito-León et al.).

The cognitive phenotype is broadened beyond traditional domains. Using a virtual-reality rod-and-frame test, one study found that older adults exhibited greater errors at selected frame orientations and a directional asymmetry not seen in younger adults, suggesting age-related changes in visuospatial processing through dissociable mechanisms of multisensory integration and lateralized attention (Adamski et al.). Additionally, a large UK Biobank cohort showed that chronic regional and widespread pain were associated with increased dementia risk, with stronger associations for widespread pain, highlighting the relevance of chronic symptom burden and pain distribution for long-term cognitive outcomes (Fu R. et al.).

Functional autonomy is a key emphasis. A cross-lagged study demonstrated that poorer cognition predicted greater subsequent dependency, especially in instrumental activities of daily living (IADL) (Fu X. et al.). A separate mediation analysis in patients with neurocognitive disorders and subjective memory complaints found a significant indirect association between comorbidity and diagnostic severity through IADL, while education also correlated with severity (Valente et al.). These findings support integrating functional autonomy, comorbidity, and educational background into multidimensional assessment and care planning.

Methodological innovation is prominent. EEG, eye-tracking, and virtual reality are used to move beyond single summary scores. Notably, Philippen et al. employed an immersive VR home-finding task to assess entorhinal cortex-dependent path integration in patients with mild cognitive impairment (MCI), older healthy controls, and younger adults. By removing distal and proximal cues, the task isolated path integration from landmark-based navigation. MCI patients showed significantly greater angular errors than age-matched controls, with a tendency for larger absolute distance errors, while older controls were impaired on all measures relative to younger adults. Increasing triangle size amplified distance but not angular errors, and scaling did not affect group differences. These findings suggest that VR-based path integration may serve as a sensitive behavioral marker for early detection of MCI and Alzheimer's disease (Philippen et al.). A study protocol for acupuncture in post-COVID-19 cognitive dysfunction uses a three-arm RCT (verum, sham, and waitlist) with multimodal MRI, including diffusion tensor imaging and resting-state functional MRI, to evaluate both efficacy and neural mechanisms (Luo et al.).

Intervention and rehabilitation also receive attention. A randomized study of 114 older adults with MCI or mild Alzheimer's dementia compared mindfulness-based stress reduction combined with reminiscence therapy vs. reminiscence therapy alone. The combined intervention improved cognitive function, depressive symptoms, behavioral and psychological symptoms, and spiritual wellbeing, supporting integrated cognitive-psychosocial approaches (Zhang F. et al.). However, not all pharmacological efforts succeed: the negative finding with pioglitazone in the CMF model cautions against extrapolating neuroprotective effects across different etiologies and underscores the need for rigorous preclinical evaluation (Alhowail).

In sum, Volume II demonstrates that interdisciplinary cognitive research is moving toward a more integrated understanding. Cognitive impairment occurs across diverse neurological, clinical, and iatrogenic conditions; environmental exposures interact with molecular and microbiome pathways; sensory, perceptual, and somatic factors contribute to the broader phenotype; cognitive decline is closely linked to functional autonomy; spatial navigation deficits may serve as early behavioral markers; and emerging technologies—including immersive VR and multimodal neuroimaging—provide objective multidimensional measures. This volume extends Volume I by placing greater emphasis on connections among clinical conditions, environmental and molecular mechanisms, functional outcomes, and multimodal assessment and intervention. Future progress will require continued integration across neuroscience, clinical medicine, psychology, public health, rehabilitation, and engineering, ultimately enabling better risk stratification, earlier detection, individualized intervention, preservation of independence, and improved quality of life for affected individuals and their families.

We sincerely thank all authors, reviewers, and editorial teams for their contributions. We hope that the studies presented in *Panoramic view of cognitive impairment: interdisciplinary cognitive research, volume II* will encourage further interdisciplinary collaboration and provide a foundation for continued efforts to understand and address cognitive impairment across the life course.
